# Hourglass-Like Constriction of the Brachial Plexus in an Adult Patient: A Case Report

**DOI:** 10.1227/neuprac.0000000000000157

**Published:** 2025-08-08

**Authors:** Ignazio Marcoccio, Jacopo Maffeis, Carolina Civitenga, Adolfo Vigasio

**Affiliations:** Hand Surgery and Microsurgical Peripheral Nerve Reconstruction Unit, Istituto Clinico Città di Brescia—San Donato Hospital Group, Brescia, Italy

**Keywords:** Brachial plexus, Hourglass-like constriction, Neuralgic amyotrophy, Neurolysis, Parsonage-Turner syndrome

## Abstract

**BACKGROUND AND IMPORTANCE:**

Parsonage-Turner syndrome is a rare disorder characterized by sudden onset of severe pain in the upper limb, followed by muscle weakness or atrophy, and remains a challenge for clinicians. Although the etiology remains unknown, surgical identification of nerve torsions and recent advances in diagnostic imaging, particularly high-resolution ultrasound and MRI, have introduced a distinct entity known as hourglass-like constriction (HLC), which may be a manifestation of Parsonage-Turner syndrome. This case report presents the first-known case of HLC involving the brachial plexus in an adult patient.

**CLINICAL PRESENTATION:**

A 66-year-old man developed brachial plexus palsy after arthroscopic rotator cuff surgery, initially manifesting as severe pain and later, after pain relief, progression to paralysis of the deltoid, biceps, and muscles innervated by the radial nerve. Despite initial conservative treatment, minimal recovery was observed at 6 months, which warranted surgery. Exploration showed a severe fibrous thickening of the anterior division was found, revealing an HLC. The unsalvageable nerve portion was resected, and direct suture was performed. Complete recovery of the deltoid nerve (M5) and almost complete recovery of the radial and musculocutaneous nerves (M4+ and M4, respectively) were noted at 30 months.

**CONCLUSION:**

The case highlights the importance of considering HLC in cases of idiopathic brachial plexus palsy, even when imaging does not exhibit clear torsions. Surgery should be considered especially if there is no spontaneous recovery after 6 months. The choice of surgical technique should depend on the severity of the constriction and the expertise of the surgeon.

**ABBREVIATIONS:**
HLC: hourglass-like constriction
NA: neuralgic amyotrophy
PTS: Parsonage-Turner syndrome.

Parsonage-Turner syndrome (PTS), also known as neuralgic amyotrophy (NA), is a rare disorder, often of unknown etiology, presenting with sudden onset of severe pain localized to the upper limb, followed by muscle atrophy.^1^ The surgical observation of nerve constriction or torsion (described as hourglass-like constriction, HLC) in patients identified as affected by NA, and the introduction of high-resolution peripheral nerve diagnostic tools such as ultrasound and MRI, have changed the therapeutic approach to this entity. Since the first description, several authors have described HLC affecting different peripheral nerves,^2-6^ with only one reported case involving the brachial plexus in a pediatric patient.^7^ We present, to our knowledge, the first case of HLC involving the brachial plexus, specifically the anterior division of the upper trunk, in an adult patient.

## CLINICAL PRESENTATION

**FIGURE 1.**
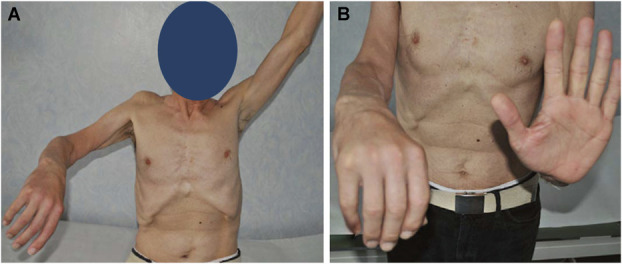
**A** and **B**, Preoperative clinical presentation of the patient with complete paralysis of the axillary, musculocutaneous, and radial nerves of the right upper extremity.

In 2016, a 66-year-old male patient consulted our unit for a right brachial plexus palsy. The patient previously underwent arthroscopic surgery for rotator cuff pathology by lateral decubitus without any issues in the immediate postoperative period. Approximately 10 days after surgery, he reported the onset of a severe pain lasting about 10 days with a subsequent full regression, followed by complete paralysis of the deltoid, biceps brachii, and of all the muscles innervated by the radial nerve (Figure 1). Clinical examination showed no signs of sensory impairment of the upper extremity. The patient was initially treated with a 1-month course of oral corticosteroids and then subjected to a scheduled follow-up. Three months after the development of the palsy, an electromyography was obtained and showed evidence of axonal regeneration of the axillary and radial nerves, without any sign of recovery instead of the musculocutaneous nerve. MRI evidenced a clear inflammation of the superior trunk and its anterior and posterior divisions.

**FIGURE 2.**
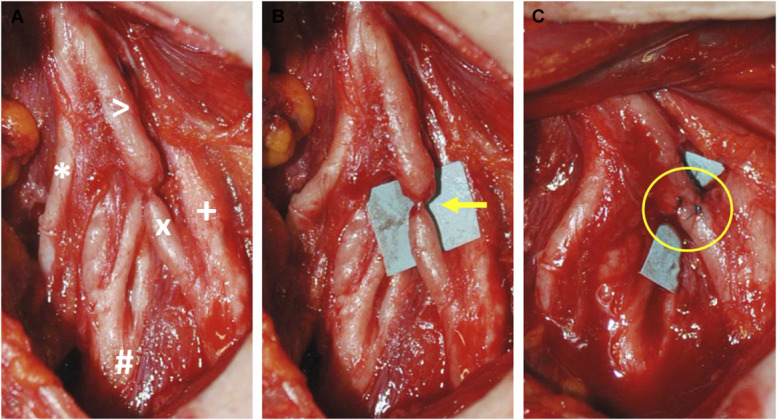
Intraoperative photographs. **A**, A severe fibrous reaction of both the upper and middle primary trunks is seen during surgical exploration. Upper trunk (>); suprascapular nerve (*); middle trunk (+); anterior division of the upper trunk (X); posterior division of the upper trunk (#). **B**, Extensive neurolysis was performed, and an hourglass-like constriction was found at the anterior division of the upper trunk (arrow). **C**, The hourglass-like constriction was resected, and a direct end-to-end suture was performed (circle).

The onset of paralysis preceded by pain led to an early suspicion of HLC. At 6 months, the recovery of the deltoid and the muscles innervated by the radial nerve was extremely slow and minimal with no recovery of the musculocutaneous nerve. In the absence of improvement, surgery was performed 7 months after paralysis. Examination of the plexus revealed a severe fibrous reaction in both the upper and the middle trunks, with a palpable hard thickening of the anterior division of the upper trunk. Extensive external neurolysis was performed on both trunks. At the site of thickening, neurolysis revealed the presence of a nerve twist compatible with HLC in a portion of fibers of the anterior division, where the nerve was almost completely compromised over 80% of its diameter. The injured portion of the nerve was resected, and a direct end-to-end suture was performed (Figure 2). The decision to proceed with surgery, despite the lack of evidence of clear nerve injury on MRI, was based on the patient's age and the very limited evidence of recovery of the axillary and radial nerves 6 months after the paralysis. In addition, the complete lack of recovery of the musculocutaneous nerve supported the choice. Four months after surgery, we observed complete recovery of the deltoid muscle (M5) and signs of fast improvement of the radial nerve, which achieved almost complete recovery (M4+) approximately 8 months after surgery.

**FIGURE 3.**
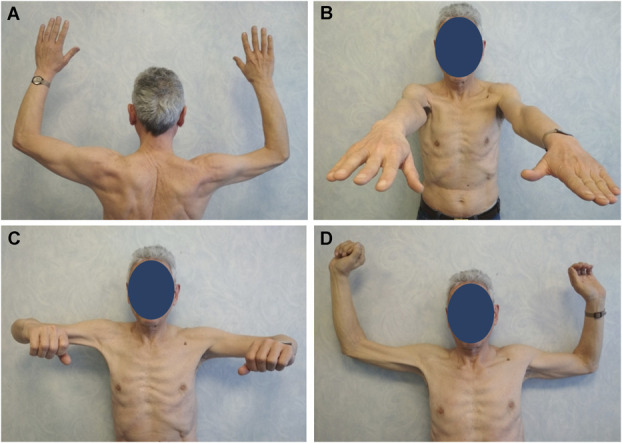
Postoperative clinical pictures. **A**-**D**, A complete recovery of the shoulder muscles innervated by the axillary nerve was achieved (M5). The radial nerve and the musculocutaneous nerve recovered almost completely (M4+ and M4, respectively) with the biceps brachii remaining slightly hypotrophic.

At 8 months, the musculocutaneous nerve also showed an improvement assessable with an M3. At 30 months postoperatively (final follow-up), the biceps regained almost full functional recovery (M4), although it remained slightly hypotrophic (Figure 3).

The patient provided informed consent for participation in the research study and consented to the publication of his images. Case reports do not need IRB/ethics committee approval at our Institution.

## DISCUSSION

Since its first description by Parsonage and Turner^8^ in 1948, NA still represents a diagnostic and therapeutic challenge. The etiology is controversial and unclear. PTS has traditionally been considered a self-limiting condition, with conservative treatments resulting in favorable long-term outcomes in 80% to 90% of cases. Reinnervation typically begins within 3 to 12 months, and full recovery may take 2 to 3 years. However, recent studies report that 25% to 60% of patients with NA have incomplete recovery, do not recover, or face recurrence.^9^

The change of approach in the management of idiopathic paralysis attributed to PTS occurred when, in 1976, Englert^10^ identified a focal nerve constriction, later defined as an hourglass constriction. Subsequently, several descriptions of HLCs affecting the peripheral nerves appeared in literature, leading to the assumption that HLC may be either the underlying mechanical cause of muscle paralysis or a PTS/NA variant.^11^ In addition, the etiology of HLC remains controversial.

Nagano and Lundborg suggested that fascicular constriction may result from local inflammation, leading to stiffening of the nerve fascicles, making them less flexible and adaptable to bending forces.^2,12^ The introduction of high-resolution peripheral nerve diagnostic tools has allowed to accurately identify cases of focal nerve torsion, giving the surgeon a precise target to address and to the patient the opportunity to benefit from an additional treatment strategy, namely surgery.^13,14^ In fact, if HLC is found and recovery is not apparent, prompt surgical exploration is suggested.^2,15^ Conversely, in cases where no constriction is detected and no healing has occurred within a reasonable time, or if regeneration remains inadequate or too slow, the watchful waiting period may be extended unless the paralysis has lasted longer than 6 to 9 months.

In the case we presented, the MRI performed 3 months after the paralysis did not show a clear torsion but rather signs of extensive inflammation of both primary trunks, so considering that the electromyography showed signs of initial axonal regeneration of the axillary and radial nerves, it was decided to extend the observation period up to 6 months. After 6 months, due to the poor recovery and the lack of biceps contraction, we decided to proceed with surgery without repeating the instrumental study. As is often the case with HLC, these are often not easily identified on initial visual inspection. The injured nerve seemed hard on manual inspection and extensive external neurolysis was required, which revealed a clear HLC.

In our experience, the degree of torsion seems greater at the level of the epineurium, but once epineurotomy is performed, the nerve damage seems more compressive than torsional. For this reason, we believe that nerve derotation does not address the compression.

In the case of isolated enlargement or mild constriction, neurolysis is indicated (if more than 25% of the nerve diameter is preserved); in a complete constriction, resection and direct neurorrhaphy is recommended. In borderline cases, we generally opt for the neurorrhaphy.^9,15^ Time factor is another crucial element, as we believe that a slight constriction can become more severe over time, thus changing the surgical technique to be adopted. In the case we presented, the constriction was so severe that a simple neurolysis was not indicated. The lack of a consistent recovery 6 months after the onset of paralysis and the patient's age were other contributing factors for the decision. A resection of the HLC and a direct tension-free suture was therefore the solution undertaken.

## CONCLUSION

This case demonstrates that even in cases of paralysis that are not purely traumatic, such as PTS/NA, the decision-making process regarding surgery should follow the same cautious timelines that are typically applied to traumatic paralysis. Surgeons should be prepared to proceed with surgery even if there is evidence of slow recovery, particularly if the underlying mechanism, the type of nerve involved, the location of the injury, and the age of the patient suggest that prolonged recovery may compromise muscle reinnervation.
